# Advanced models for respiratory disease and drug studies

**DOI:** 10.1002/med.21956

**Published:** 2023-04-29

**Authors:** Jesus Shrestha, Keshav Raj Paudel, Hojjatollah Nazari, Vivek Dharwal, Sajad Razavi Bazaz, Matt D. Johansen, Kamal Dua, Philip M. Hansbro, Majid Ebrahimi Warkiani

**Affiliations:** ^1^ School of Biomedical Engineering University of Technology Sydney Sydney New South Wales Australia; ^2^ Centre for Inflammation Centenary Institute and University of Technology Sydney Sydney New South Wales Australia; ^3^ Discipline of Pharmacy, Graduate School of Health University of Technology Sydney New South Wales Australia; ^4^ Faculty of Health, Australian Research Centre in Complementary & Integrative Medicine University of Technology Sydney Ultimo New South Wales Australia; ^5^ Institute for Biomedical Materials and Devices, Faculty of Science University of Technology Sydney Ultimo New South Wales Australia

**Keywords:** animal models, drug discovery, lung‐on‐a‐chip, medicinal research, respiratory diseases

## Abstract

The global burden of respiratory diseases is enormous, with many millions of people suffering and dying prematurely every year. The global COVID‐19 pandemic witnessed recently, along with increased air pollution and wildfire events, increases the urgency of identifying the most effective therapeutic measures to combat these diseases even further. Despite increasing expenditure and extensive collaborative efforts to identify and develop the most effective and safe treatments, the failure rates of drugs evaluated in human clinical trials are high. To reverse these trends and minimize the cost of drug development, ineffective drug candidates must be eliminated as early as possible by employing new, efficient, and accurate preclinical screening approaches. Animal models have been the mainstay of pulmonary research as they recapitulate the complex physiological processes, Multiorgan interplay, disease phenotypes of disease, and the pharmacokinetic behavior of drugs. Recently, the use of advanced culture technologies such as organoids and lung‐on‐a‐chip models has gained increasing attention because of their potential to reproduce human diseased states and physiology, with clinically relevant responses to drugs and toxins. This review provides an overview of different animal models for studying respiratory diseases and evaluating drugs. We also highlight recent progress in cell culture technologies to advance integrated models and discuss current challenges and present future perspectives.

## 1 INTRODUCTION

The morbidity and mortality related to respiratory diseases impose substantial socioeconomic burdens on individuals and societies worldwide.^1^ Five respiratory conditions predominantly contribute to the global health burden; chronic obstructive pulmonary disease (COPD), asthma, tuberculosis, lung cancer, and acute respiratory infections.^2^, ^3^ The common risk factors for chronic respiratory diseases include exposure to indoor and outdoor pollutants, tobacco use, allergens, occupational exposure, obesity, physical inactivity, and an unhealthy diet.^4^ Increased exposure to these risk factors and the aging of populations have caused substantial increases in the prevalence and disease burden of respiratory diseases worldwide. The ongoing global coronavirus disease 2019 (COVID‐19) pandemic, caused by severe acute respiratory syndrome coronavirus‐2 (SARS‐CoV‐2), is still a major global crisis with over 317 million confirmed cases and 5.51 million deaths.^5^ Disastrous bushfires and wildfires worldwide have also increased exposure to hazardous levels of air pollution with deleterious respiratory impacts.^6^

With a dire need to address the growing global burden of respiratory diseases, rapid and efficient preclinical evaluation models are urgently required to fast‐track the development of safe and efficacious new therapeutics and vaccines.^7^, ^8^ Some of these therapeutics target specific components of pulmonary disease such as inflammasomes,^9^ interleukins (ILs),^10^ matrix metalloproteinases (MMPs),^11^ and mitochondrial dysfunction.^12^ Drug development is an extensive, intricate, and expensive process, and has high attrition rates in clinical trials.^13^ The average cost of research and development for taking a new product to the clinic is estimated to be $2.5 billion and takes up to 15 years.^14^ Despite immense collaborative efforts and increased expenses to identify and develop the most effective and safe drugs, few are approved for clinical trials.^7^ Compared to cardiovascular, neurological, and other diseases, the drugs approved for use in pulmonary medicine are few, and there are fewer drug candidates to address their growing healthcare burden.^15^ To streamline drug development and limit costs, it is essential to eliminate ineffective drug candidates as early as possible, which can be achieved by utilizing new preclinical screening approaches with high efficiency and accuracy.^14^

Laboratory‐based preclinical and ex vivo, and in vitro drug models that use different cell types and cell‐based assays are commonly employed by the pharmaceutical industry and research laboratories to assess drug candidates.^16^, ^17^ Although these models are simple to use, can be well controlled, and offer high throughput for basic screening and testing of drugs, they fail to mimic the in vivo tissue architecture, physiology, and cellular interactions in human cells and tissues and are unable to predict complex drug metabolism processes and adverse reactions.^18^ Thus, advanced human‐relevant models that mimic the human lung microenvironment along with tissue–tissue interfaces, chemical gradients, and mechanical factors are urgently required to better model respiratory diseases and expedite pulmonary preclinical research.

**Figure 1:**
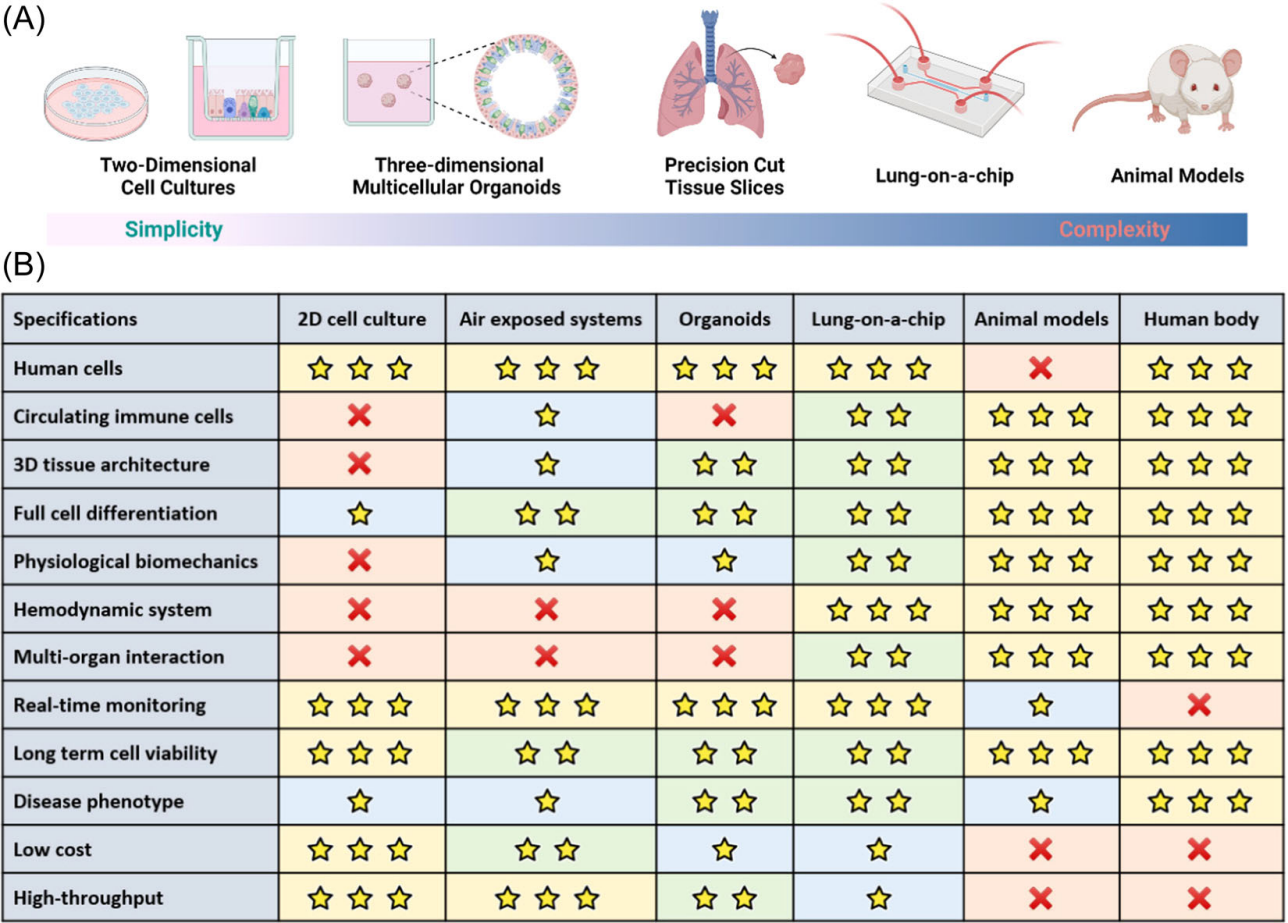
Cell culture models of human lung: (A) Commonly used cell culture models to study respiratory diseases. (B) Comparison of features of existing cell culture models. 2D, and 3D cell culture models, and animal models are extensively used to preclinically assess drugs in the drug development process. Recently, organoids and microfluidic organ‐on‐a‐chip models have gained significant attention owing to their 3D physiologically relevant environment. All these models have their advantages and limitations. Models that mimic complex human physiology with clinically relevant and reproducible responses are required to identify the most effective and safe drugs. (*Note*: Animal models can incorporate human cells, e.g., human‐derived xenografts.). [Color figure can be viewed at wileyonlinelibrary.com]

Here, we overview the existing ex vivo and in vitro platforms that are used to study human lung pathophysiology and host‐respiratory pathogen interactions (Figure 1). We summarize recent advances in technologies and cell culture platforms that replicate the in vivo environment to provide physiologically relevant clinical data. Additionally, we discuss the applications of these models in different pathologies and their use for screening drugs to alleviate the global burden of respiratory diseases.

## 2 ADVANCED RESPIRATORY DISEASE ANIMAL MODELS

A plethora of animal models has been developed to investigate the pathogenesis and study potential treatments for respiratory diseases. This includes the use of mice, rats, ferrets, and guinea pigs, with mice being by far the most popular.^19^, ^20^ The use of animal models depends upon their availability, ease of housing, breeding, manipulation, and cost. In the context of respiratory diseases, these animal models can be broadly categorized into three groups based on the techniques used to induce the features of human disease: (A) antigen‐induced models (e.g., ovalbumin (OVA)‐induced allergic asthma, cigarette smoke‐induced COPD^21^, ^22^); (B) spontaneous models (e.g., lung fibrosis model in the *viable motheaten* mouse linked with an increased level of tumor necrosis factor‐alpha (TNF‐α)^23^); and (C) genetically modified models (e.g., genetically engineered *Kras* ^*LSL‐G12D*^ mouse model of lung cancer^24^).

### 2.1 Asthma

Allergens such as OVA, house dust mites (HDM), cockroaches, air pollution, and biomass smoke are used as a stimulant to trigger asthma in mouse models.^25^, ^26^, ^27^ OVA (from chicken eggs) is a well‐established experimental antigen used to investigate the pathophysiologic changes during asthma. Key features of the OVA model are an increase in eosinophils in bronchoalveolar lavage fluid (BALF), airway mucus hypersecretion, and airway hyperresponsiveness (AHR).^28^ A recent study by Kim et al. found that OVA increases BALF eosinophil peroxidase and nitric oxide linked to serum IL‐4 and IL‐13.^21^ Chronic challenges also induce airway fibrosis and remodeling.^29^

HDM is another allergen used in murine asthma models. Exposure of BALB/c mice intranasally to HDM extract (25 μg in 10 μl of saline) acutely or for several weeks results in severe and persistent lung inflammation with an accumulation of CD4 + lymphocytes and overproduction of helper T cell Type 2‐related cytokines by splenocytes,^25^, ^30^ airway remodeling with the longer models and AHR.

Cockroach allergen is also a potent stimulator of asthma features. Cockroach allergen‐immunized mice can develop fatal asthma‐like attacks. In this model, mice are initially sensitized with i.p injection (50 µl) and subcutaneous injection (50 µl) of 120,000 protein nitrogen unit/ml of cockroach allergen followed by challenge with intranasal instillation (20 µl) on Days 7, 14, 20, and 22. Asthma features include overproduction of mucus, increase in serum immunoglobulin E (IgE), infiltration of leukocytes, increased eosinophil in BALF, impaired lung function revealed by decreased pulmonary compliance, and increased airway resistance.^31^ Other animal models of asthma‐induced pathogens such as *Alternaria alternata fungus*, viruses such as *Chlamydia, Haemophilus influenza, and influenza A*, and the respiratory syncytial virus are listed in Table 1.

**Table 1:** Experimental models of respiratory disease.

Disease	Inducer/mouse model	Features	References
Asthma	OVA‐induced in BALB/c mice	Eosinophilia, increased eosinophil peroxidase, IL‐4, IL‐13, nitric oxide, mucus hypersecretion, AHR, airway remodeling with chronic exposure	^21^, ^29^
	HDM induced in BALB/c mice	Severe and persistent lung inflammation with the accumulation of CD4 + lymphocytes and overproduction of helper T cell Type 2‐related cytokines by splenocytes, airway remodeling with chronic exposure	^25^, ^30^
	Cockroach allergen‐induced Abr‐/‐ mice with FVBJ inbred background	Infiltration of leukocytes, increased eosinophil in BALF, increased serum IgE, mucus hypersecretion, impaired lung function with decreased pulmonary compliance, and increased airway resistance	^31^
	Fungus (*Alternaria alternata*) induced in BALB/c mice	This includes predominant eosinophilic Th2 responses in wild‐type mice and neutrophilic in *Il4, Il13*, and *Stat6* ^*‐/‐*^ mice. The neutrophilic responses in *Stat6* ^*‐/‐*^ mice characterized by increased lung levels of CXCL1, TNF‐α, CXCL2, and CXCL5	^32^
	Ambient air PM induced in BALB/c mice	Upregulation of mRNA expression of TNF‐α in lung tissue	^33^
	Diesel exhaust particle‐induced in BALB/c mice	Increased eosinophil, IL‐5, IL‐17F, and CCL20, decreased levels of IFN‐γ in BALF and AHR	^33^
COPD	CS‐induced in BALC/c and C57BL/6 mice	Chronic airway and lung inflammation with increased macrophages and neutrophils in BALF, expression of TNF‐α, CxCl2, KC, and IL‐1B in the lung, airway remodeling, and fibrosis, alveolar enlargement/emphysema, impaired lung function, and gas exchange	^22^, ^34^
	Porcine pancreatic elastase‐induced in BALB/c and C57BL/6 mice	Increase neutrophils and macrophage and TNF‐α, IL‐6, KC, GCSF, MCP‐1 in BALF	^35^, ^36^
	Porcine pancreatic elastase‐induced in BALB/c mice	Increased mRNA expression of TNF‐α, IL‐6, GCSF, MIP‐2, KC, MMP‐2, and MMP‐9 and protein expression of p65 NF‐KB in lung tissue homogenates, alveolar enlargement/emphysema	
	LPS induced in guinea pigs	Inflammation of the airway, hyperplasia of goblet cells, edema, persistent bronchoconstriction	^37^, ^38^
	LPS‐induced C57BL/6 mice	Neutrophil‐dependent emphysematous changes in lung architecture associated with apoptosis	
	Biomass (80 g of dried dung) smoke (1 h per day for 1 month) in rabbits	Respiratory epithelial proliferation, alveoli destruction, emphysematous changes	^39^
Lung cancer	CS‐induced in A/J and Swiss mice	Formation of pulmonary adenomas	^40^
	NNK induced in A/J mice	100% develop lung cancer with various doses of NNK for 8 weeks and 9–19 weeks of rest	
	NNK induced in the golden hamster	80% of golden hamsters developed lung cancer with 0.012 mmol NNK, three times weekly for 25 weeks, total dose = 0.91 mmol	^41^, ^42^, ^43^, ^44^
	NNK induced in F344 rats	90% develop lung cancer at age 108 weeks when administered 5.0 ppm NNK daily in drinking water starting at 8 weeks of age	
	NNK induced in ferrets	Develop both preneoplastic and neoplastic lesions in lungs with 50 mg/kg body weight NNK once a month for 4 months, followed by rest periods of 24, 26, and 32 weeks	
	Diethylnitrosamine induced in FVB/N mice	70% develop lung cancer with a single high dose of 15 μg/g body weight; intraperitoneally followed by 1‐year rest	^45^
Lung fibrosis	Bleomycin induced in WT or *Fblnlc* ^*‐/‐*^ C57BL/6 J mice	Significant collagen deposition with intranasal administration of 1 dose of bleomycin sulfate at 0.05 U/mouse followed by 4 weeks of rest	^46^
	HDM induced in WT or *Fblnlc* ^*‐/‐*^ C57BL/6 J mice	Significant collagen deposition with intranasal administration of 25 μg in 30 μl of sterile saline for 5 days/week for 5 weeks	^46^
	CS (12 research cigarettes, twice a day, 5 times per week for 4, 6, 8 weeks) exposure in WT or *Fbln1c* ^*–/–*^ C57BL/6 J mice	Increased fibulin‐1 and airway fibrosis with increased collagen deposition around small airways after 8 weeks	^46^
	Silica induced in C57BL/6 mice	Neutrophilic inflammation and fibrotic changes, including dense fibrotic foci with interstitial fibrosis	^47^
	Crocidolite or Libby amphibole asbestos‐induced in WT C57BL/6 J mice	Augmented pulmonary fibrosis and apoptosis in cells at the bronchoalveolar duct junctions after intratracheal installation of 100 μg/50 μl of crocidolite or Libby amphibole asbestos followed by 3 weeks or 2 months rest	^48^
COVID‐19	K18‐hACE2 mice	Significant weight loss and rapid onset of the disease are characterized by high clinical scores. High viral titers in the lung and brain. Extensive lung inflammation and pulmonary decline. Severe but tractable disease phenotype makes this model attractive for vaccine and drug discovery	^49^
	AdV and AAV	Mild weight loss and clinical scores in wild‐type mice. High viral replication in the lung during the early stages of infection rapidly decreases with disease progression. Increased severity in immunocompromised mice that is dependent on the knockout line used (e.g., IFNAR). This model is advantageous for understanding genetic determinants of SARS‐CoV‐2 infection dependent on the mouse line used; however, is not suitable for drug or vaccine studies.	^50^
	Mouse‐adapted SARS‐CoV‐2	Rapid and significant weight loss and clinical score progression, with high lung viral titers. Extensive lung inflammation closely mimics the disease severity of the K18‐hACE2 mouse model. Useful animal model to examine vaccines and drugs; however, as the virus has been adapted to mice, there is the potential that infection may not recapitulate all aspects of human COVID‐19.	^51^, ^52^
	Ferrets	Mild weight loss and clinical disease, high viral lung replication and shedding in nasal washes, saliva, urine, and feces, early fever onset. The high similarity of the respiratory tract between ferrets and humans, with vaccine and drug efficacy studies often used in ferrets as a preclinical model.	^53^, ^54^, ^55^
	Hamsters	Moderate weight loss with the minimal clinical score. High viral replication in the lung, with extensive lung inflammation and pulmonary damage. Used extensively in the preclinical development of vaccine and drug candidates for COVID‐19.	^56^, ^57^
	NHP	The gold standard for preclinical animal models. Moderate weight loss with progressive clinical scores. High viral replication in the lower respiratory tract with severe lung inflammation. Used in the final stages of vaccine and drug efficacy studies before human clinical trials.	^58^, ^59^, ^60^, ^61^

Abbreviations: AAV, adeno‐associated virus; Abr, active BCR‐related gene; AdV, adenovirus; AHR, airway hyperresponsiveness; BALF, bronchoalveolar lavage fluid; CCL20, chemokine (C‐C motif) ligand 20; CD, a cluster of differentiation; COPD, chronic obstructive pulmonary disease; Fbln1c, fibulin/c; GCSF, granulocyte‐macrophage colony‐stimulating factor; HDM, house dust mite; IFN‐ɤ, interferon; IgE, immunoglobulin E; IL, interleukin; KC, keratinocytes‐derived chemokine; LPS, lipopolysaccharide; MCP‐1, monocyte chemoattractant protein‐1; MIP‐2, macrophage inflammatory protein 2; MMP, matrix metalloproteinase; mRNA, messenger RNA; NHP, nonhuman primates; NNK, nicotine‐derived nitrosamine ketone; OVA, ovalbumin; PM, particulate matter; SARS‐CoV‐2; severe acute respiratory syndrome coronavirus‐2; STAT6, signal transducer and activator of transcription 6; TNF‐a, tumor necrosis factor‐alpha; WT, wild type.

### 2.2 COPD

Cigarette smoke is the principal risk factor for COPD.^62^, ^63^ Mice chronically exposed to cigarette smoke also develop the hallmark features such as BALF neutrophilia and emphysema.^64^, ^65^, ^66^, ^67^ We developed a short‐term model of experimental COPD by exposing wild‐type BALB/c or C57BL/6 mice to the smoke from 12 research‐grade cigarettes (3R4F) twice per day, 5 days/week for 8–12 weeks using nose‐only exposure.^22^, ^68^ This is equivalent to a pack‐a‐day human smoker.^19^ The COPD features observed in these mice are significant increases in the chronic airway and lung cellular and cytokine/chemokine (increased TNF‐α, CXCL2, IL‐1B, keratinocytes‐derived chemokine (KC)/murine equivalent of human IL‐8) inflammation, airway remodeling and fibrosis, emphysema and impaired lung function and gas exchange compared to control mice exposed to normal air.^22^, ^34^ IL‐22 and its receptors are also increased in the same model of experimental COPD,^69^ while Toll‐like receptor‐2 was protective.^70^ Other models use whole‐body exposures that take 6–12 months to induce these features.^71^

Other mouse models are induced with elastase (cleaves collagen and elastin). Porcine pancreatic elastase (1U/mouse) to generate COPD by intratracheally installing it into BALB/c and C57BL/6 mice.^68^, ^72^ Mice were killed after 1 day, 3 days, or 21 days of elastase treatment. Although this is not the exposure that induces COPD in humans, features of COPD do develop. These include increased airway and neutrophil and macrophage counts and cytokines (TNF‐α, IL‐6, KC, granulocyte‐macrophage colony‐stimulating factor (GCSF), monocyte chemoattractant protein‐1 (MCP‐1), were significantly increased on Day 3. In addition, there were increased mRNA levels of TNF‐α, IL‐6, GCSF, macrophage inflammatory protein‐2 (MIP‐2), KC, and protein expression of nuclear factor kappa‐light‐chain‐enhancer of activated B cells (NF‐κB) in lung tissue and the destruction of airway spaces (a feature of emphysema).^35^ Another study observed that elastase (1U/mouse, intratracheal instillation, followed by 1–3 weeks rest) increased the mRNA expression of MMP‐2 and MMP‐9 key enzymes involved in the cleavage of extracellular matrix (ECM).^36^

Lipopolysaccharide (LPS) is an endotoxin of Gram‐negative bacteria.^73^ LPS can be detected in the BALF of patients with COPD, which has led to speculation of roles for it in the progression of the disease.^74^, ^75^ It can be administered alone or with other challenges such as cigarette smoke to induce COPD features in mice.^76^ LPS administration induces hallmark features of airway inflammation such as excessive mucus production, narrowing of the bronchial lumen, and chemotaxis of neutrophils and macrophages to airway spaces.^77^, ^78^ LPS is administered intratracheally into mice at a dose of 5 μg/installation/mouse^77^ or 0.1 ml of a 100 mg/ml LPS solution in normal saline for rats.^78^ This dose is equivalent to LPS entering the human lung after smoking approximately 25 cigarettes.^79^ In guinea pigs and mice, the chronic damage in the lungs induced by LPS is associated with the overexpression of TNF‐α and IL‐18.^37^

Human exposure to biomass smoke for a prolonged period is another cause of chronic respiratory disease.^80^ Compared to unexposed healthy people, those who are exposed have an odds ratio of 2.44 for developing COPD.^81^ Thus, another animal model of COPD is in vivo exposure to varying doses and periods of biomass smoke. Rabbits exposed to biomass smoke by burning 80 g of biomass‐dried dung 1 h/day for a month had increased proliferation of respiratory epithelial cells and significant alveolar destruction compared to air‐exposed controls.^39^ Other animals such as C57BL/6 mice and Sprague–Dawley rats are exposed to biomass smoke to induce COPD.^82^ This suggests that people cooking indoors with dung in low‐ and middle‐income countries may develop COPD features after repeated exposure.^83^

Although animal models are widely used to model COPD, in contrast to the variable pathology and different stages of COPD severity in humans, the existing animal models are confined to mimic only a few (but not all) characteristic features of human COPD which must be taken into account when investigating the clinical application of these models. Therefore, the assessment of animal models and the interpretations of results need to be based on the alignment of the characteristic features of human COPD to advance the understanding of disease pathogenesis.^84^

### 2.3 Lung cancer

Different strains of wild‐type mice have differing susceptibilities to cigarette smoke or tobacco carcinogen‐induced lung cancer. A/J and Swiss SWR strains are considered susceptible, while BALB/c are intermediate, and C57BL/6, C3H, DBA, and B6C3F1 are relatively resistant.^85^ A/J and Swiss mice are genetically susceptible to lung cancer when exposed to whole‐body cigarette smoke (reference cigarette 2R4F) for 5 months with a 4‐month rest period. There were significant increases in the incidence of lung tumors and tumor multiplicity in mice exposed to cigarette smoke.^40^ The most potent carcinogens in cigarette smoke are polycyclic aromatic hydrocarbons, tobacco‐specific nitrosamine, and Benzo [a] pyrene.^86^ These carcinogens induce different types of lung cancer. Nicotine‐derived nitrosamine ketone (NNK) induces adenocarcinoma,^87^ whereas, *N*‐nitroso‐tris‐chloroethylurea promotes the development of squamous cell carcinoma.^88^ A single high dose (15 μg/g body weight; intraperitoneally) of diethylnitrosamine followed by 1‐year rest is enough to induce lung cancer in 70% of FVB/N mice.^45^ The primary tumors express the cytokeratin‐7 characteristic of epithelial neoplasms and thyroid transcription factor‐1 specific to the thyroid and lung adenocarcinoma. No mutations were observed in *KRAS* and epidermal growth factor receptor(*EGFR)* genes while mTOR was highly activated.^45^

Several transgenic mouse models develop features that resemble those of human lung cancers. Among them are mice deficient in tumor suppressor genes such as tumor suppressor protein 53 (*Trp53*), mutated in colorectal carcinoma protein (*Mcc*), retinoblastoma protein (*Rb*), phosphatase and tensin homolog (*PTEN*), and RNA‐binding protein 5 (*Rbm5*) or mice with mutation of proto‐oncogenes such as Kirsten rat sarcoma virus (*Kras*), tyrosine kinase receptors (*Trk*), extracellular signal‐regulated kinase (*Erk*), *Wnt* and *Myc*.^85^, ^89^

Ferrets are also used to model lung cancer. Intraperitoneal injection of NNK in ferrets (50 mg/kg body weight) once a month for 4 months, followed by postexposure rest periods of 24, 26, and 32 weeks led to the development of both preneoplastic and neoplastic lesions in the lungs.^41^ The preneoplastic lesions were characterized by squamous metaplasia and dysplasia, and atypical adenomatous hyperplasia. The major discovery in this study was the high expression of nicotinic acetylcholine receptors on broncoepithelial cell membranes.^41^ Urethane is another potent tobacco carcinogen used in mice models to induce lung adenocarcinoma. A recent study of C57BL/6 mice injected with 1 g/kg urethane intraperitoneally for 10 consecutive weeks followed by 14 weeks of rest showed the development of lung adenocarcinoma. This study revealed that airway epithelial cells were more susceptible to urethane‐induced KrasQ61R mutations than alveolar type II cells, suggesting that tobacco‐induced lung adenocarcinoma is initiated in airway epithelial cells.^90^ Collectively, these studies show that mouse models can be used to investigate genetic mechanisms and discover drugs for lung cancer.^91^

### 2.4 Lung fibrosis

Lung fibrosis is the primary feature of idiopathic pulmonary fibrosis (IPF) and an important feature of asthma and COPD.^92^ Bleomycin is commonly used to induce pulmonary fibrosis in mouse models. Other inducers of lung fibrosis are HDM extract, silica, cigarette smoke, and asbestos. Although mice are preferred, other animals such as rats, hamsters, rabbits, guinea pigs, dogs, and primates are also used in lung fibrosis models.^93^, ^94^ Administration of a single dose of bleomycin (intranasal with 0.05U/mouse) with or without 4 weeks of rest in one mouse model and treatment with HDM extract (intranasal with 25 μg in 30 μl of sterile saline) for 5 days/week for 5 weeks in another resulted in significant airway inflammation and collagen deposition around the airways.^46^ The airway inflammation and remodeling in both bleomycin and HDM‐treated mice were dependent on the ECM protein fibulin‐1c activation of TGF‐β.^25^, ^46^ Knockout of the Fbln1c gene and targeted inhibition of Fbln1c could decrease collagen deposition in the airway and protect against AHR.^25^

Administration of asbestosis to animals can replicate the pathologic changes that occur in human pulmonary fibrosis in asbestosis. For animal models, asbestos should only be used after baking to avoid contamination with LPS and to avoid LPS‐related inflammatory changes.^95^ A single dose of asbestos fibers delivered via intratracheal administration can induce pulmonary fibrosis, although it may take more than a month to develop the characteristic features, such as collagen deposition, oxidative stress, fibroblastic foci, and alveolar epithelial cell injury.^96^ However, such models have limitations; fibrosis is mostly central but not subpleural, and collagen deposition is unequally distributed around the lungs. Pulmonary fibrosis due to the deposition of asbestos fibers is due to apoptosis of alveolar epithelial cells followed by the polarization of macrophages to M2 phenotype and increased production of pro‐fibrotic cytokines by activated T lymphocytes.^97^, ^98^

The chronic instillation of silica into rat lungs results in the formation of fibrotic nodules, which are similar to those in the lungs of exposed humans.^99^ In mice, the nature of the experimental silicosis that develops depends on the dose of silica, route, length of exposure, and mouse strain.^100^ Silica can be administered in mice via inhalational/aerosolization,^100^ oropharyngeal aspiration,^47^ or intratracheal delivery.^101^ Comparatively, C3H/HeN, MRL/MpJ, and NZB mice are more prone to developing aerosolized silica‐induced fibrosis than BALB/c mice which have little fibrotic response.^100^ In intratracheal silica administration, the C57BL/6 strain is more susceptible than CBA/J mice.^102^ After administration, silica accumulates in the lung and induces persistent inflammatory responses followed by the development of fibrotic nodules around silica deposits. Intratracheal models take 2–4 weeks to develop,^47^ whereas inhalation induces disease that closely resembles human silicosis but takes 1–6 months.^101^

### 2.5 COVID‐19

SARS‐CoV‐2 is the causative agent of COVID‐19. It causes severe viral infection resulting in excessive inflammatory responses driven by a pro‐inflammatory cytokine storm syndrome, which results in extensive airway inflammation.^50^, ^103^ The ongoing COVID‐19 pandemic has driven the rapid discovery of effective vaccines and drugs. To develop and test novel therapeutics, preclinical animal models are essential to facilitate the translational investigation of candidate drugs and vaccines and expedite human implementation.^49^, ^104^ Various small animal models with mice, ferrets, and golden hamsters are commonly used to study COVID‐19 pathophysiology and identify the most effective therapeutics.^20^ However, the most critical aspect to consider is selecting an appropriate animal model where the SARS‐CoV‐2 Spike (S) protein binds to the viral entry receptor, Angiotensin‐converting enzyme 2 (ACE2).

Wild‐type mice are not permissive to the ancestral SARS‐CoV‐2 infection due to incompatibility between the S protein and mouse ACE2 (mACE2). Consequently, several genetic strategies have been implemented to overcome this barrier to produce mouse models that are an excellent preclinical platform for COVID‐19 research. The most common approach utilizes transgenic mice expressing human ACE2 (hACE2) under the control of the human cytokeratin promotor (K18), which is localized exclusively in epithelial cells of the lung, gastrointestinal tract, liver, kidney, heart, and brain.^105^ This mouse line, K18‐hACE2, was originally developed to study the pathophysiology of SARS‐CoV, responsible for the first SARS epidemic in 2003. However, as both SARS‐CoV and SARS‐CoV‐2 enter host cells via binding of the S protein to hACE2, K18‐hACE2 mice are highly susceptible to infection with both with significant weight loss, extensive lung inflammation, high viral lung titers, and uniform lethality with relatively low viral inocula.^106^ Interestingly, K18‐hACE2 mice also develop extensive neurological inflammation with high viral titers during the late stages of SARS‐CoV‐2 infection. However, the mechanisms of how this occurs are unknown, which is a caveat with this severe mouse model.^107^ Due to the severe and uniform lethality of the K18‐hACE2 mouse model, it has been used extensively for vaccine development and preclinical testing. Accordingly, Counoupas et al. found that a single dose of BCG‐adjuvanted COVID‐19 vaccine provides sterilizing immunity against SARS‐CoV‐2 in K18‐hACE2 mice, resulting in no recoverable viral titers in the lung, reduced pro‐inflammatory cytokine production, and no histological lung inflammation. Furthermore, the targeted antibody response generated by BCG‐based COVID‐19 vaccination in mice effectively neutralized the B1.1.7 and B.1.351 variants of SARS‐CoV‐2.^49^ Therapeutic administration of Remdesivir and Plitidepsin, both antiviral inhibitors of SARS‐CoV‐2 replication, was protective in reducing lung viral titers and lung inflammation in K18‐hACE2 mice.^108^ Additionally, monoclonal antibody therapy at both the early and late stages of SARS‐CoV‐2 infection rescued mice from lethal infection.^109^, ^110^

Alternative approaches involve the use of adenovirus (AdV) or adeno‐associated virus (AAV) transduction systems to transiently induce expression of hACE2 in recipient cells or following intranasal administration to mice to induce SARS‐CoV‐2 infectivity in the absence of endogenous hACE2.^110^, ^111^ AdV and AAV systems are advantageous as they can utilize readily available genetically modified mouse lines without the need to backcross to K18‐hACE2 mice. However, both AdV and AAV SARS‐CoV‐2 mouse models result in only mild weight loss, clinical presentation, and lung inflammation when challenged with high viral inocula in wild‐type; however, they both support high viral replication during the early stages of infection when hACE2 is abundantly expressed.^110^, ^111^ While the use of AdV and AAV mouse models is useful in understanding the genetic determinants of COVID‐19, they are relatively ineffective in testing novel therapeutic agents and vaccines.

Another approach is to mouse adapt SARS‐CoV‐2. This involves the continual passage of highly virulent and human‐specific SARS‐CoV‐2 through mice, followed by the recovery of infectious virus to repeat the process and facilitate adaptation of SARS‐CoV‐2 to the mouse host. This has resulted in mouse‐adapted SARS‐CoV‐2 variants that cause significant weight loss, clinical scores, and severe lung inflammation, which recapitulates wild‐type SARS‐CoV‐2 in K18‐hACE2 mice.^51^, ^52^ However, the continuous passage of SARS‐CoV‐2 through mice to induce evolutionary pressure on the virus to mutate and adapt to the host may cause unknown and potentially deleterious genetic changes, which may deviate from the original SARS‐CoV‐2 variant and question the relevance of the mouse‐adapted SARS‐CoV‐2 model. Interestingly, mouse‐adapted SARS‐CoV‐2 models have been used to test the efficacy of preclinical vaccine candidates and the endogenous administration of IFN‐λ, both of which reduce viral replication and protect mice from severe disease.^51^

Other animal models that are employed to investigate the pathophysiology of SARS‐CoV‐2 include ferrets and hamsters, although these are less commonly used compared to traditional mouse models. Both ferret and hamster ACE2 have a high degree of homology with hACE2, thus enabling SARS‐CoV‐2 infection in these animals.^112^

Finally, nonhuman primates (NHP) are considered the “gold standard” as preclinical animal models for understanding the pathophysiology of COVID‐19. However, they are much less commonly used than smaller animal models due to high costs and unique facility requirements, as well as ethical considerations. Rhesus Macaques and Cynomolgus Macaques develop moderate disease phenotypes with moderate weight loss and clinical scores, extensive pulmonary infiltration, and high viral titers in nasal and throat swabs, as well as BAL.^58^, ^113^ NHPs have been used in vaccine development and efficacy studies as a final stage preclinical test before human trials. Vaccines protect Rhesus Macaques from viral replication in the lower respiratory tract, decrease lung inflammation and induce the development of high‐neutralizing antibody titers.^59^, ^60^ Antiviral drugs such as Remdesivir and monoclonal and neutralizing antibodies also significantly mitigate clinical disease features, reduce lung inflammation, and lower viral lung titers in Rhesus Macaques.^61^, ^114^

Although animal models are widely used for respiratory disease‐specific investigations, preclinical drug development, and testing, they also come with several limitations. In some cases, they do not reproduce the structural, mechanical, and functional properties of the human tissue and thus fail to mimic the inherently complex nature of tissues and organs. Moreover, promising discoveries and treatments in animal models do not necessarily prove to have favorable outcomes for humans.^115^ Thus, advanced models like organs‐on‐chip are required to decrease the need for both traditional 2D cell culture methods and animal studies. These advanced models are described in detail in the following section.

## 3 LUNG BIOPSIES AND TISSUES IN RESPIRATORY RESEARCH

Animal models of different lung diseases are valuable in increasing our understanding of lung development, pathophysiology, and diseases; the inherent differences between animal and human lung physiology make it imperative to develop human lung model systems. These models complement and are complemented by in vivo animal models. Clinical biopsies of the airways or lungs are collected and cells or tissues are extracted for examination.^62^ Bronchoscopies are performed to collect airway brushings for epithelial culture, and airways pinch biopsies can also be obtained.^65^, ^116^ Primary airway epithelial cells are cultured at the air–liquid interface (ALI) and differentiate into differentiated columnar epithelium with the basal, club, goblet, and ciliated cells. These cultures are valuable in pathogenesis, drug, and infection (e.g., influenza, respiratory syncytial virus, SARS‐CoV‐2) studies. In asthmatic individuals, airway biopsies are performed to study different phenotypes of severe asthma to direct treatments.

Lung biopsies are performed to ascertain an accurate diagnosis of the onset of interstitial lung disease or confirm the absence/presence of cancer. Various lung biopsy procedures include needle, thoracoscopic, transbronchial, and open biopsy.^117^, ^118^ Many studies have utilized airway and lung tissues obtained by a biopsy to study the pathogenesis and effects of treatments in the context of asthma, COPD, lung fibrosis, and cancer.^92^, ^119^, ^120^, ^121^

Airway and lung biopsies contain all of the cell and tissue compartments affected by respiratory diseases, including the airways and the lung parenchyma. The samples obtained by biopsy can be snap frozen (to study the gene expression and proteins levels of different markers of asthma, and also epithelial cells can be resurrected from them for ALI culture), paraffin‐embedded (to assess histological changes like goblet cell hyperplasia/metaplasia and immune cell infiltration in distal and central airways), and processed for high magnification imaging (scanning electron microscopy [SEM] and transmission electron microscopy [TEM]).^122^ Biopsies are also helpful in diagnosing respiratory conditions. They can be used to differentiate severe asthma from allergic bronchopulmonary aspergillosis, autoimmune airway disease, and hypersensitivity pneumonitis.^119^ However, since asthma is primarily an airway disease, BAL and sputum are more widely used than bronchial biopsies.^123^ In COPD patients, inflammation occurs primarily in the small airways and lung parenchyma and all airway lung samples are important.^124^ Indeed COPD‐linked structural changes and expression of inflammatory proteins can be studied utilizing bronchial and lung biopsies and their use provides insights into COPD pathogenesis.^125^ Stefan et al. reported increased NF‐κB expression in bronchial biopsies from smokers and patients with COPD. NF‐κB is a redox‐sensitive transcription factor that regulates multiple pro‐inflammatory pathways.^126^ Additionally, decreased histone‐deacetylase activity has been reported in bronchial biopsies that explain the limited effectiveness of steroids in individuals with COPD.^127^ Many studies have used lung biopsies and tissues to study COPD, such as defining the roles of microRNAs in driving pathogenesis through a SATB1/S100A9/NF‐κB pathway, and the role of necroptosis.^65^, ^128^ IPF is a chronic, progressive, and fibrotic lung disease where healthy lung tissue is permeated with excessive ECM, resulting in reduced lung function.^92^ Transbronchial lung biopsy and lung cryobiopsy are used to diagnose IPF and to study disease pathogenesis and progression.^129^

Although airway and lung biopsies offer an accurate and detailed method to study the pathogenesis and effects of treatments in different lung diseases, there are multiple challenges associated with this approach. Obtaining enough tissues to perform multiple analyses is challenging. Also, the patients that undergo lung biopsy may have complications of hemoptysis, pulmonary venous air embolism, pulmonary hemorrhage, and pneumothorax.^130^, ^131^ Thus, optimizing the use of airway and lung biopsies for studying different respiratory diseases is needed.

Resected lung tissues from severe COPD, IPF, and lung cancer patients can be used for the interrogation, manipulation, or extraction of cells for further analysis (Table 2).^65^, ^128^ They are valuable in defining pathogenic mechanisms and testing therapies compared to healthy control tissues from failed lung transplants. Tissues from the lungs as far as possible from lung tumors can also be used for healthy or COPD studies depending on the diagnosis of the donors. These tissues have some obvious confounding factors.

**Table 2:** Recent studies utilizing lung biopsies/tissue in respiratory research.

Disease	Study model	Significance	References
COPD	Resected lung lobes, lung transplant from COPD individual, and healthy controls	Pathogenic stem cell variants dominating COPD lungs contribute to metastasis, inflammation, fibrosis, and airway obstruction	^132^
	Lung tissue of patients with COPD and healthy controls	Role of MiR‐223 in cigarette smoke‐linked pulmonary inflammation	^133^
	Lung tissue of patients with COPD and control subjects without COPD	Role of necroptosis in COPD‐linked inflammation, airway remodeling, and emphysema	^65^
	Lung biopsy samples from 50 soldiers with postdeployment constrictive bronchiolitis, 8 patients with sporadic constrictive bronchiolitis, 55 patients with COPD, and 25 healthy control subjects	Role of T‐helper cell type 1‐type adaptive immune response in airway wall remodeling in constrictive bronchiolitis	^134^
	Lung tissue samples from patients with COPD at different stages	Comparison of the inflammatory response, protease‐antiprotease balance, oxidative stress, and apoptosis in different groups	^135^
	Lung tissue of patients with COPD and control subjects without COPD	Role of MAP3K19 in COPD pathogenesis	^136^
Lung fibrosis	Lung tissues from non‐IPF donors and IPF patients undergoing lung transplantation	Role of Lipofibroblasts in lung fibrosis	^137^
	Control lung tissue collected from patients undergoing surgery for cancer. Lung fibrosis tissue collected from patients undergoing biopsy	Role of Synergistic role of HSP90α and HSP90β in lung fibrosis	^138^
	Control lung tissue samples from lobectomies. IPF samples obtained from surgical lung transplant explants	Role of dysfunctional lactate metabolism in human alveolar type II cells in the context of lung fibrosis	^139^
	Flash‐frozen lung tissue from 94 human subjects with IPF and 83 control subjects	Role of miR‐199a‐5p in tissue fibrosis	^140^
Asthma	Epithelial brushings and BAL	Role of 15 lipoxygenase 1 in glutathione redox changes in asthma	^141^
	Airway biopsies from patients with severe asthma present	Role of Oncostatin M in bacteria‐induced inflammation/mucous production in severe asthmatics	^142^
	Bronchial epithelial cells obtained by bronchoscopy from healthy controls, asthmatics (different stage)	Metabolic differences in bronchial epithelial cells from asthmatics compared to healthy controls	^143^
	Bronchoalveolar lavage cells from asthmatics and healthy controls	Role of lung iron levels in asthma pathogenesis	^144^

Abbreviations: BAL, bronchoalveolar lavage; COPD, chronic obstructive pulmonary disease; IPF, idiopathic pulmonary fibrosis.

## 4 ORGANOIDS

Organoids, or miniaturized organs, are intricate multicellular clusters produced during in vitro culture of stem cells related to a specific organ.^145^ Stem cells start to form organoids via innate developmental self‐assembly and form complex tissue‐like structures and simplified versions of organs in 3D microenvironments.^146^ The recent progress in stem cell manipulation techniques has led to the development of in vitro culture systems mimicking the native microstructure of organs to produce functional and relevant cellular complexes.^147^ Further improving the systems by generating organoids with vessel‐like structures that are vital for the transportation of nutrients and waste is a demanding concern. The lack of nutrient transportation to the inner sides of these cellular complexes can limit the survival, proliferation, differentiation, and functionality of organoids.^148^

Recently, researchers have generated different organoid models for regeneration,^149^ disease modeling,^150^ cancer,^151^ and pharmacologic studies^152^ of the respiratory system (Table 3). The lung‐relevant organoids are generated by coculturing different somatic, progenitor, and stem cells. To generate organoids for each part of the lung, from the airways to the alveoli, the complicated 3D architecture, ECM, fluid dynamics of the microenvironment, cell composition, and intracellular interactions of that specific parts need to be recapitulated.^153^ Ideally, the vascularized and complicated regions of the alveoli would also be recapitulated. There have been substantial recent advances in lung organoid studies that we review and highlight.

**Table 3:** Recent studies utilizing lung organoids in respiratory research.

Stem cell source	Disease	Organoids	Features of lung organoids	References
hESC	Different human lung diseases	Lung organoid	In vitro: Alveolar type 2 cells and immature alveolar type 1 cells	^154^
			In vivo: Short term: lung progenitor cells	
			Long term: lung distal bipotent progenitor cells, AT2 cells, and immature AT1 cells, mesenchymal cells, vasculature, neuroendocrine‐like cells, and nerve fiber structures	
hPSCs	Different human lung diseases	Lung organoids (Modeling epithelial‐mesenchymal crosstalk)	In vitro (50–85 days): Bronchi/bronchioles of the developing human airway surrounded by lung mesenchyme in organoids	^155^
			In vitro (22 days): Cells in organoids were transcriptionally similar to human fetal bud tip progenitors	
		Bud tip progenitor organoids (exploring epithelial fate decisions)	Organoids have in vivo engraftment potential	
Genetically‐engineered human ESCs	(Fibrotic lung disease)	Hermansky–Pudlak Syndrome mutant fibrotic organoids	The upregulation of interleukin‐11 was observed in epithelial cells of mutant fibrotic organoids (common in type 2 alveolar epithelial cells in end‐stage idiopathic pulmonary fibrosis)	^156^
	Hermansky–Pudlak Syndrome associated‐interstitial pneumonia			
Genetically‐engineered human ESCs	Human/viral vector interactions	Lung bud organoids	The recombinant adeno‐associated virus showed tropism for the human lung parenchyma (day 59 in vitro)	^157^
			Testing drugs for SARS‐CoV‐2	
Distal Lung Epithelial Cells	The therapeutic effect of MSCs on lung	Bronchiolar and alveolar organoids	Mesenchymal stem cell coculture with organoids enhances their differentiation	^158^
hiPSCs	Respiratory viral disease (parainfluenza) in infant's lung	Lung organoids	Parainfluenza virus caused viral shedding without morphological changes	^159^
			Respiratory syncytial virus infection induced detachment and shedding of infected cells into the lung organoid lumens	
Human iPSCs or human ESCs	Pulmonary disease modeling	Lung organoids	Organoids containing epithelial, mesenchymal cells, and ciliated cells	^160^
			Produce of surfactant was observed in organoids	
Induced‐alveolar epithelial type 2 cells (hiPSCs)	Alveolar disease modeling	Mature alveolar organoids (Alveolospheres)	Epithelial‐only alveolospheres express lamellar bodies	^161^
			Serial passage of alveolospheres does not need mesenchymal support	
hiPSCs‐derived SFTPC^+^ alveolar stem cells	Drug toxicology models	Alveolar organoids	Heterogeneous alveolar epithelial type II cells found in organoids	^162^
			Stable expansion and long‐term culture observed in organoids	
Lung cancer resections and metastasis biopsies	Respiratory syncytial virus infection modeling	Human airway organoids	Basal cells, multiciliated cells, mucus‐producing secretory cells, and CC10‐secreting club cells are formed in organoids	^163^
			Organoids derived from cystic fibrosis biopsies recapitulate lung central disease characteristics	
			Viral infection causes significant epithelial remodeling in organoids	
Lung biopsies	Epithelial remodeling in lung disease	Bronchospheres organoids	Organoids do not need permeable support for proliferation and can contain functional multiciliated cells, mucin‐producing goblet cells, and airway basal cells	^164^
CRISPR/Cas9 genome (Transcription factor Grainyhead‐like 2 (Grhl2)‐) edited‐primary human basal cells	Lung epithelial modeling	Lung organoids	Loss of Grhl2 inhibits organoid morphogenesis	^165^
		Polarized mucociliary epithelium		

Abbreviations: hESC, human embryonic stem cell; hiPSCs, human induced pluripotent stem cells; hPSCs, human pluripotent stem cells.

### 4.1 Stem cell‐derived lung organoids

Lung organoids can be generated using mesenchymal (MSCs), embryonic (ESCs), and induced pluripotent (iPSCs) stem cells. The study of stem cell‐derived organoids can increase our understanding of developmental processes and underlying pathophysiology to treat lung disease and injuries. ESCs were the first stem cells to be used and were isolated from embryos. They have been used in cell biology studies for over 30 years.^166^ These stem cells have many attributes for studying cellular behavior and signaling during the developmental process in the ESC period. The self‐organization of ESCs is a critical point in organoid biology and is genetically encoded inside cells leading to the formation of biological structures.^167^ They have been implemented in the production of lung organoids for biomedical applications. Chen et al. generated 3D lung organoids by differentiating human ESCs toward immature airway epithelial cells. Their study showed that the transplantation of hESCs‐derived organoids into the subcapsular pocket of the kidney of immune‐deficient mice improved the growth of MSC cells, vascularization phenomena, secretions, and nerve regeneration in transplanted areas.^154^ In another study, Miller et al. established a protocol for differentiating human pluripotent stem cells (hPSCs) into human lung bronchial and bronchiolar organoids.^155^ Strikoudis et al. generated lung‐engineered mutations related to Hermansky–Pudlak syndrome (HPS) in genes of human ESCs and differentiated them into lung organoids to model the pathogenesis of HPS‐associated pulmonary fibrosis. Their study showed that IL‐11 expression by alveolar type II cells increased inside these fibrotic organoids.^156^ In a recent study, Meyer‐Berg et al. generated human transduced lung bud organoids using gene‐edited human ESCs for pulmonary gene therapy applications. They modeled the transduction of recombinant AAV from the luminal surface of organoids via inhalation to develop new ways of testing therapeutics for SARS‐CoV‐2.^157^

**Figure 2:**
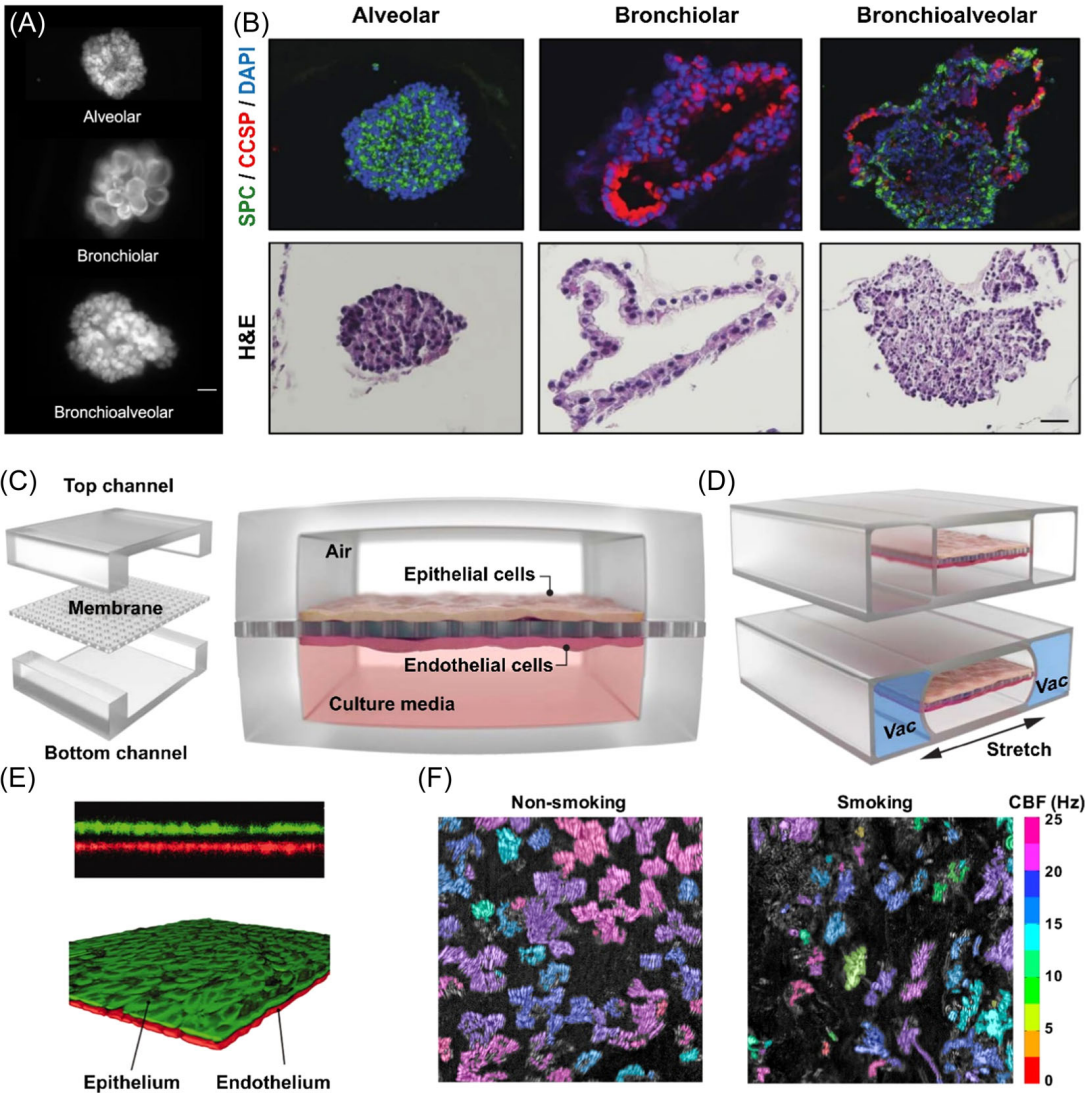
Different models of airway organoids and lung‐on‐a‐chip: (A) Representative Green fluorescent protein (GFP^+^) images of alveolar, bronchiolar, and bronchioalveolar/mixed organoids formed from coculture of mesenchymal (MSCs) with distal lung epithelial cells. Scale bar: 50 μm. (B) Immunofluorescence staining for Surfactant Protein‐C (SPC‐ green) and Clara cell secretory protein (CCSP ‐red) (top row) and Hematoxylin/Eosin (HE) images (bottom row) on the different types of organoids. DAPI: blue stained nuclei. Scale bar: 50 μM. (C) Expanded view of the lung‐on‐a‐chip model with an ECM‐coated membrane separating two chambers with human alveolar epithelial cells in one and capillary endothelial cells cultured on either side of the porous membrane. (D) Flexible sidewalls and a central membrane with adherent cells were stretched regularly by the application of a cyclic vacuum to the two lateral hollow chambers. (E) Monolayers of alveolar epithelium (stained with CellTracker Green) and microvascular endothelium (stained with CellTracker Red) cultured inside lung‐on‐a‐chip device. (F) On‐chip time‐lapse images of cilia beating of the bronchiolar epithelial culture in the absence or presence of cigarette smoke. (A) and (B) reproduced with permission from Leeman et al.^158^ (C) and (D) from Park et al.,^169^ (E) from Huh et al.,^170^ and (F) from Benam et al.,^171^ respectively. [Color figure can be viewed at wileyonlinelibrary.com]

MSCs are multipotent stem cells resident in some parts of adult tissues, including the umbilical cord, bone marrow, and adipose tissue.^168^ These types of stem cells can proliferate, self‐renew and differentiate into various tissues. The addition of biological elements such as signaling molecules, exosomes, and extracellular vesicles can increase their differentiation toward desired cells in lung organoids. In this respect, a study by Leeman et al. shows that coculturing lung progenitor cells with MSCs significantly increases the formation of bronchiolar and alveolar organoids. These cells also enhance the alveolar differentiation and self‐renewal ability of lung epithelial cells^158^ (Figure 2A,B).

iPSCs are a new generation of stem cells that have attracted much recent attention in cell therapy, disease modeling, and pharmacological studies.^172^ These cells are directly generated via genetically reprogramming somatic cells. In the next steps, the cells with stemness are derived from donors and can differentiate into different cells needed for modeling organs. iPSCs technology also removes the ethical issues associated with the clinical and research application of ESCs.^173^ Several groups have used iPSCs to generate organoids for different organs including the cerebrum,^174^ kidney,^175^ liver,^176^ and lung.^177^, ^178^ For the respiratory system, Protto et al. generated lung organoids by differentiating hESCs and iPSCs and modeled the infections of respiratory virus infection in the lungs in children and infants. They observed the formation of mesoderm, pulmonary endoderm, branching airway, and alveolar structures in these organoids. They were then infected with parainfluenza viruses to model respiratory viral disease.^159^ In another study, Leibel et al. generated 3D multicellular organoids containing epithelial, MSC, and ciliated cells and alveolar macrophages via differentiation of hESCs and hiPSCs to model infectious disease.^160^ Jacob et al. also generated iPSCs by editing surfactant protein C genes in donor stem cells and differentiated them into granulous pneumocytes (alveolar type II cells) to form alveolar organoids.^161^ Alveolar type II cells produce pulmonary surfactants and play an important role in gas exchange.

The dysfunctionality of alveolar type II cells is fatal in lung diseases, especially lung cancer, which is a huge clinical issue. Yamamoto et al. generated alveolar organoids using hiPSCs and expanded them to model human alveolar development and study the in vitro toxicity of drugs.^162^ In a recent study related to the 2020 COVID‐19 pandemic, Bose et al. generated 3D human lung organoids and 3D bronchial transient epithelial/progenitor cells by differentiating ethnicity‐based iPSCs. These organoids and cells modeled the lower respiratory tract, providing researchers a way to understand differences in severity and infectivity of SARS‐CoV‐2.^179^ The high costs of generating, gene editing, purifying, and expanding iPSCs are significant limitations and need advanced technology facilities.

### 4.2 Patient‐derived lung cancer organoids

Organoids derived from the cancerous lung of patients can mimic the native architecture of tumors and are considered the optimal in vitro models for anticancer drug studies.^180^ Kim et al. produced lung cancer organoids recapitulating the histology of five lung cancers. They isolated cancer cells from patients with squamous cell carcinoma, adenocarcinoma, adenosquamous carcinoma, small cell carcinoma, and large cell carcinoma to generate organoids. These organoids responded differently to anticancer drugs as each drug targets a specific genetic alteration in a tumor. Cancer organoids with breast cancer 2 genes, EGFR, and EGFR‐mutant/MSC‐epithelial transition (MET)‐amplified mutations responded better to laparib, erlotinib, and crizotinib, respectively.^181^ In another study, Sachs et al. generated human airway organoids from lung cancer resections and metastasis biopsies for drug screening studies.^163^

### 4.3 Normal lung‐derived organoids

Various organoids have been generated by culturing cells isolated from different regions of normal Lungs such as the trachea, airways, and nasal epithelium. The main elements of these organoids are basal progenitor cells, airway secretory cells such as goblet cells, alveolar type II cells, multiciliate cells, and Alveolar type 2 cells.^164^ Hild et al. developed lung organoids termed mature bronchospheres composed of functional multiciliate, airway basal, and mucin‐producing goblet cells for high‐throughput studies of human airway epithelium.^164^ Lung organoids can also be used to identify crucial genes that control airway function, such as fluid/gas transport, selective permeability, innate immunity, and barrier formation using clustered regularly interspersed short palindromic repeats (CRISPR)/CRISPR‐associated protein 9 (Cas9) gene‐editing technology.^182^ The findings can be useful in predicting possible oncogene‐activated mutations introduced by CRISPR/Cas9 and cancer in lung organoids. Recently, different groups have reported the use of primary lung alveolar organoids to better understand the underlying pathogenesis of SARS‐CoV‐2 infections and identify effective drugs against the virus.^183^, ^184^

Despite its widespread usage, controlling and reproducing the biochemical and biophysical environment required for organoid development is challenging. The lack of dynamic vascular supply and dependence on passive diffusion for growth make it difficult to grow large organoids. Additionally, the variations in size, structure, and gene expression among organoids restrict their application in drug testing and disease modeling. However, altering the inherent properties of cells provides a potent means of enhancing the stability of organoids and customizing them for specific uses. The field of genetic engineering, personalized organoids, and bioprinting organoids and organoid‐forming cells are still in its early stages, but they hold the potential to advance the use of organoids in tissue engineering and facilitate the creation of functional organs and the large‐scale cultivation of synthetic tissues.

## 5 LUNG‐ON‐A‐CHIP MODELS

Recent advances in tissue engineering combined with additive manufacturing techniques have given rise to organs‐on‐a‐chips, miniaturized dynamic in vitro cell culture models that recapitulate the vital structural and functional elements of human organs.^185^, ^186^ These systems recreate the tissue‐tissue interface, vascular perfusion and biochemical gradients, and infusion of immune cells and other connective tissues.^187^ Primary cells, iPSCs, or patient‐derived organoids can be used to line the device with a continuous flow of media in the endothelium‐lined channels resulting in an active exchange of nutrients, drugs, and waste.^188^ Creating an entire human lung with all the critical structural and functional elements is not technically feasible.^186^ Lung‐on‐a‐chips offer the ability to reproduce the well‐defined functional units of the lungs. They can mimic the mucociliary barrier of the airways, alveolar‐capillary interface, and inflammatory responses, which is critical to studying the disease process and evaluating new drugs and toxins.

### 5.1 Early models

The lung‐on‐a‐chip sector has gained substantial traction in terms of its number and design following the model of Huh et al., which aimed to mimic the human lung environment more closely.^189^ Numerous comprehensive reviews have extensively discussed lung‐on‐chip models, including the study of different sections of the human respiratory system (alveolus, bronchi), fabrication processes, cell types (e.g., primary cells, co‐/triple cultures), and use of different materials (polydimethylsiloxane [PDMS], poly(methyl methacrylate), bio‐ink).^185^, ^189^, ^190^, ^191^ Takayama and his group developed a small human airway‐on‐a‐chip and cultured primary lung epithelial cells at the ALI for >3 weeks to form a structurally intact fully differentiated airway epithelium with barrier functions.^192^ An automatic microfluidic plug generator was integrated into this model to precisely reproduce the propagation and rupture of liquid plugs similar to mucus plugs found in diseased lungs. They showed that the plug propagation and rupture generated deleterious fluid mechanical stresses leading to cellular‐level injury under flow conditions that caused symptoms observed in different pulmonary diseases.^192^

The first multicompartment model of the lung‐on‐a‐chip model was designed by Huh et al. with PDMS using soft lithography‐based microfabrication techniques.^170^ Their model had two flow channels separated by an ECM‐coated thin porous and flexible membrane (Figure 2C). The upper and lower channels were lined with human alveolar epithelial cells and human lung capillary endothelial cells, respectively, on the porous membrane. The resulting barrier tissue mimicked the alveolar‐capillary interface in vivo. Once confluent, the alveolar cells were cultured at the ALI by aspirating the medium from the upper channel, whereas media was infused continuously in the lower channel. A cyclic vacuum was applied to the two lateral hollow chambers to stretch the flexible sidewalls regularly (Figure 2D,E). Along with stretching walls and the central membrane, the adherent cell layers with the ECM were also stretched. Their novel design replicated the dynamic mechanical deformation of the alveolar‐capillary interface during physiological breathing patterns.

Their design reproduced the complex integrated organ‐level responses when the alveolar epithelium is exposed to toxic nanoparticles and bacteria. When pathogenic bacteria were inserted in the alveolar compartment of the chip, inflammatory cytokines were released by the epithelium and the activation of the microvascular endothelial cells on the adjacent compartment led to the expression of high levels of adhesion molecules. Circulating primary human neutrophils in the endothelial channel established firm adhesion to the activated endothelium and transmigrated across the alveolar‐capillary barrier. Furthermore, their model identified the adverse effects of the cyclic mechanical strain from physiological breathing patterns on inflammatory and toxic responses of the lung when exposed to silica nanoparticles. In another study, the same model was used to reproduce the development and progression of toxicity‐induced pulmonary edema in cancer patients treated with IL‐2.^193^

Stucki et al.^194^ developed a breathing lung‐on‐a‐chip model to mimic the alveoli. Their design included a stretchable micro diaphragm to deform the alveolar‐capillary barrier, demonstrating the effects of mechanical strain induced by physiological breathing patterns on barrier permeability and interactions between cells and the ECM. A vertically stacked model with three culture compartments was micro‐engineered by Sellgren et al.,^195^ which used cocultured primary human airway cells with lung fibroblasts and microvascular endothelial cells that exhibited barrier function. Fishler et al.^196^ developed models using bifurcated microchannels with bifurcating alveolar ducts mimicking alveolar morphology to study the complex alveolar airflow patterns and determine the dispersion and deposition of inhaled hazardous or pharmaceutical aerosols. Advanced models of lung‐on‐a‐chip that better mimic the characteristic alveoli network and its microenvironment are being fabricated using different hydrogels and membranes.^197^, ^198^

### 5.2 Disease modeling and drug testing

After establishing numerous models and with further advances in fabrication techniques, several groups have applied the lung‐on‐a‐chip system to model different aspects of lung physiology, respiratory diseases, toxicological analysis, and pathophysiology. Benam et al. developed a human small airway‐on‐a‐chip to model human lung inflammatory disorders, asthma, and COPD and evaluated therapeutic responses.^199^ IL‐13 perfusion through the vascular channel of the chip led to significant airway remodeling with hypersecretion of inflammatory cytokines, goblet cell hyperplasia, and decreased cilia beat frequency, similar to that observed in asthmatic airways. This asthmatic phenotype was suppressed by treatment with an anti‐inflammatory drug, Tofacitinib (Janus kinase inhibitor). The chip mimicked key disease features using primary airway cells derived from COPD patients, showing selective cytokine secretion, increased recruitment of neutrophils, and characteristics of acute exacerbations on exposure to bacterial and viral pathogens.

The same group further utilized the small airway‐on‐a‐chip model to study the effects of cigarette smoke on the bronchial epithelium of normal lungs or COPD patients on cellular and genetic level responses in vitro by connecting the dynamic chip to a smoking instrument to mimic human smoking behavior and reproduced COPD‐specific responses^171^ (Figure 2F). Shrestha et al. utilized his simple open well design of lung‐on‐a‐chip to study the effects of cigarette smoke on epithelial cells, followed by treatment with Budesonide, an anti‐inflammatory drug.^200^ Using the model based on the original small airway chip, Villenave et al. used human Rhinovirus (HRV), a primary trigger of asthma exacerbations, to infect the chip airway that resulted in diapedesis of immune cells across the endothelium. When IL‐13 treated chips were infected with HRV, neutrophil recruitment to the vascular walls further increased, which could be pharmacologically inhibited with a C‐X‐C Motif Chemokine Receptor 2 (CXCR2) antagonist.^201^ By culturing bronchial smooth muscle cells on thin elastomeric films, Nesmith et al. fabricated a human airway muscle‐on‐a‐chip to reproduce in vitro asthmatic airway smooth muscle responses of hypercontractility and altered relaxation when exposed to IL‐13.^202^

The behavior, invasion variations, and growth patterns of human nonsmall cell lung cancer (NSCLC) in different microenvironments was studied by Hassell et al. using a lung cancer‐on‐a‐chip model.^203^ Significant suppression of the growth of tumor cells was observed when the cyclic mechanical strain was applied to the chip to mimic breathing patterns. The tumor response to Tyrosine Kinase Inhibitors (TKIs) was also tested in the device. Similarly, Yang et al. cocultured human NSCLC with human fetal lung fibroblasts in their model to test an EGFR‐targeted Antitumor drug, Gefitinib.^204^ Khalid et al. developed a lung cancer‐on‐a‐chip platform with inbuilt sensors for real‐time physiological monitoring.^205^ Trans‐epithelial electrical resistance‐based cytotoxicity evaluation of the anticancer drugs doxorubicin and docetaxel was performed. Hou et al. cocultured pulmonary epithelial cells with vascular endothelial cells in their lung‐on‐a‐chip model and exposed them to cigarette smoke extract (CSE), which induced the degradation of intercellular connections and activated the epithelial–mesenchymal transition process.^206^ CSE exposure activated proto‐oncogene signal transducer and activator of transcription 3 (STAT3), which was inhibited by HJC0152, suggesting a potential new treatment for cigarette smoke‐induced COPD and lung cancer.

### 5.3 Infections

The potential to infect lung epithelial cells with pathogens (bacteria, viruses, or fungi) in a lung‐on‐a‐chip platform provides the opportunity to study host‐pathogen interactions.^191^, ^207^ Thacker et al. developed a lung‐on‐a‐chip infection model to study the dynamics of host‐*Mycobacterium tuberculosis* interactions and the effects of pulmonary surfactant in early infection.^208^ The bacteria proliferated in the presence of surfactant, whereas its growth was much slower or inhibited in the absence of surfactant. The removal of virulence‐associated lipids and proteins from the bacterial surface demonstrated the protective role of surfactants in tuberculosis, which is critical in managing the disease. Barkal et al. studied the inflammatory responses of bronchioles to a common fungal pathogen, *Aspergillus fumigatus*, by exposing fungus into the central airway lumen of their model.^209^ The fungus traversed through the epithelium into the surrounding hydrogel. Neutrophils perfused in the vascular channel migrated toward fungal hyphae structures through the endothelium, mimicking in vivo findings. The lung‐on‐a‐chip approach was utilized by Deinhardt‐Emmer et al. to study the central pathophysiological features of pneumonia by coinfection with *Staphylococcus aureus* and influenza A virus.^210^ The model replicated the spatiotemporal spreading of the pathogens and identified potential therapeutic targets in treating pneumonia.

With the demand for effective prophylactics and therapeutics to prevent and treat rapidly spreading pandemic viral infections such as those of influenza A virus and SAR‐CoV‐2/COVID‐19, repurposing existing Food and Drug Administration‐approved drugs as antiviral preventions or therapeutics can be highly effective.^211^ Different groups have opted to explore preclinical approaches with human organ‐on‐a‐chip technology to identify the most effective drugs in a systematic and human‐relevant way.^211^ Zhang et al. modeled a human alveolar chip to study SARS‐CoV‐2‐induced pulmonary injury and immune responses at the organ level.^212^ They observed a high multiplicity of infection with high viral infection in epithelial compared to endothelial cells. The crucial role of immune cells in disrupting the alveolar barrier and exacerbating inflammation was observed as the authors reported the recruitment of immune cells, endothelium detachment, and elevated levels of inflammatory cytokines. They also showed that the antiviral, Remdesivir, reduced the disruption of the alveolar‐capillary barrier, indicating its potential to treat COVID‐19.

Using a bronchial airway‐on‐a‐chip infected with influenza A virus, Si et al. demonstrated the efficacy of the viral neuraminidase inhibitor Oseltamivir alone and co‐administration with Nafamostat, an anticoagulant drug.^213^ Nafamostat doubled the treatment‐time window of Oseltamivir. The antimalarials (Chloroquine, Hydroxychloroquine) and antiviral (Arbidol), which had shown promising results with cell lines, were unable to inhibit pseudotyped SARS‐CoV‐2 infection in the model, indicating no clinical benefits in animals and human patients. Amodiaquine and its active metabolite, desethylamodiawuine, showed promising results both in vitro and in vivo, suggesting that they may be promising therapies for COVID‐19. Findings from these studies suggest that the lung‐on‐a‐chip may be a suitable model to replicate clinically relevant organ‐level responses to viral infections. Combined with existing cell‐based and animal assays, lung‐on‐a‐chip models offer a powerful platform to study the underlying pathophysiology of respiratory diseases and expedite the discovery of effective and safer drugs to combat pandemic viral infections.^211^, ^212^ Different studies conducted using lung‐on‐a‐chip models have been summarized in Table 4.

**Table 4:** Different studies utilizing Lung‐on‐a‐chip models.

Disease studied	Model features	References
Asthma	IL‐13 induced acute exacerbation of asthma and evaluated tofacitinib, a JAK inhibitor, as a potential new therapeutic	^199^
	HRV‐16‐induced asthmatic exacerbation and assessed the efficacy of MK‐7123, a CXCR2 antagonist	^201^
	Human asthmatic musculature to mimic asthmatic bronchoconstriction and bronchodilation and evaluated HA1077, as a potential treatment for treating acute bronchoconstriction and preventing hypercontraction of bronchial smooth muscles.	^202^
COPD	Investigated the role of STAT3 phosphorylation in cigarette smoke‐associated COPD and malignant transformation of bronchial epithelium and evaluated HJC0152 as a potential treatment	^206^
	The model used epithelial cells from COPD patients and replicated the viral and bacterial infection‐led clinical exacerbation of COPD and evaluated BRD4‐inhibitor, a new anti‐inflammatory drug	^199^
	Bronchial epithelium isolated from COPD patient lungs to study the in vitro responses to e‐cigarettes	^171^
Lung cancer	Multiorgan chip to study the in vivo lung cancer metastasis	^214^
	Evaluated the effects of EGFR‐targeted Antitumor drugs: Gefitinib, Osimertinib, and Afatinib	^215^
	Effects of different patterns of hypoxia on major subtypes of lung cancer	^216^
	Replicated the lung cancer growth and invasion patterns and effects of physiological breathing motions on the pattern	^203^
Lung fibrosis	Structured color materials to explore IPF phenotypes with mechanical visuals and the role of cyclic stretching	^217^
	iPF derived fibrotic disease model for cystic fibrosis, s and studied fibroblast‐endothelium‐epithelium interactions	^218^
	Investigated CXCL12‐CXCR4 axis mediated ECP induced migration of fibrocytes towards epithelium in airway remodeling	^219^
	Cystic Fibrosis model and response to *Pseudomonas aeruginosa* and circulating polymorphonuclear leukocytes	^220^
COVID‐19	Tested FDA‐approved drugs on SARS‐CoV‐2 pseudoviral infection	^211^
	Efficacy of multiple antiviral drugs on SARS‐CoV‐2 pseudoviral infection	^197^
	Inflammatory responses and pathological changes following SARS‐CoV‐2 infection. Tested potential antiviral therapies	^212^
	Inflammatory responses and entry process of SARS‐CoV‐2 pseudovirus to explore SARS‐CoV‐2 pathogenesis	^221^
Pulmonary Thrombosis	Studied pathophysiology and tested new therapeutic	^222^
Toxins	Interleukin‐2 induced pulmonary edema and testing of potential new therapeutics Angiopoietin‐1, and TRPV4	^193^
	Nanotoxicity of TiO_2_ and ZnO nanoparticles	^223^
	Assessment of exposure to different concentrations of air pollutant fine particulate matter (PM _2.5_)	^224^
	Physiological responses to smoke generated by electronic cigarettes	^171^
	Assessed the effects of cigarette smoke and the anti‐inflammatory drug, Budesonide	^200^

Abbreviations: BRD4, bromodomain‐containing protein 4; COPD, chronic obstructive pulmonary disease; COVID‐19, coronavirus disease 2019; CXCL12, C‐X‐C chemokine ligand 12; CXCR2, C‐X‐C motif chemokine receptor 2; CXCR4, C‐X‐C chemokine receptor type 4; ECP, eosinophil cationic protein; EGFR, epidermal growth factor receptor; FDA, Food and Drug Administration; HRV‐16, human rhinovirus; IL‐13, interleukin‐13; IPF, idiopathic pulmonary fibrosis; SARS‐CoV, severe acute respiratory syndrome coronavirus‐2; STAT3, signal transducer and activator of transcription 3; TiO2, titanium oxide; TRPV4, transient receptor potential vanilloid 4; ZnO, zinc oxide.

### 5.4 Organoids‐on‐a‐chip

Organoids and organs‐on‐a‐chip are two inherently different yet complementary approaches to replicate the specific functions and major features of human organs in vitro.^169^ These two advanced approaches can be synergistically combined to recapitulate the complexities of human organs in an easily reproducible, controllable, and accessible manner.^225^ Organoids being similar to actual organs are an improved model for identifying targets and validation in the drug discovery process. In contrast, organs‐on‐a‐chip provide greater efficacy and safety testing through more controlled and reproducible results.^226^ Organoids‐on‐a‐chip offer control of the biochemical and biophysical environment that are key in developing fully mature organoids with physiological relevance and functionality. Stable flow of chemical gradients in specific time courses, implementation of mechanical forces such as shear stress, and solid mechanical forces like physiological and biomechanical cues develop a fully functional and mature organoid.^227^, ^228^ The ability to mimic perfusable blood vessels or incorporate functional vasculature in organs‐on‐chips provides the growing organoids with sufficient nutrition and oxygen for maturation and longer survival.^229^ The organs‐on‐chips platform also allows the co‐culture of different cells and tissues with the capability of replicating multiorgan physiological interactions, providing multiorganoid systems as an enhanced platform for preclinical drug screening.^169^ Organoids‐on‐chips can be used to create patient and population‐specific disease models using patient‐derived organoids, which can be applied to personalized and possibly regenerative medicine.^230^

Despite being reliable, cost‐effective, reproducible, and physiologically relevant, lung‐on‐a‐chip systems still face important challenges. PDMS, the commonly used material to fabricate organ‐on‐a‐chip devices, can interfere with pharmacological studies as it can absorb lipophilic drugs.^188^ Moreover, ECM‐coated PDMS membranes used to mimic tissue interfaces and culture media as a surrogate of blood does not precisely represent the in vivo physical and transport properties.^231^ To resolve this issue, researchers are exploring different fabrication materials and techniques such as 3D printing, micro‐milling, and solvent bonding of glass, thermoplastics, and silicon to create models capable of incorporating multiple features.^232^ Maintaining primary human cell heterogeneity is another major challenge. Significant donor‐to‐donor variation of primary human cells and organoids or even with cells from a single source impairs quality control.^233^ Commonly used cell lines in the devices do not recapitulate the tissue‐specific functions and lack adequate cell heterogeneity. The small cell numbers used in devices can also lead to limited secretions, which may not be enough to be detected by widely used traditional assays. Complex designs with multiple features are difficult to fabricate and use, leading to limited throughput. The growth of the lung‐on‐a‐chip sector has recently accelerated, as reflected by bibliometrics with the increase in the number of publications and patents over recent years. With pharmaceutical companies beginning to adopt organ‐on‐a‐chip technology, a report from Yole Développement, a marketing, technology, and strategy consulting company, expects the market to grow at a compound annual growth rate of 28.6% from $29.6 million in 2018 to $133.9 million by 2024.^234^ Although it is a new technology with numerous challenges, further integration of automation and inbuilt sensors, and international funding strategies, organ‐on‐chips technology, and its applications will grow substantially in the near future.^235^

## 6 CONCLUSION AND FUTURE PERSPECTIVES

The risk and incidence of chronic respiratory diseases continue to increase globally. The ongoing COVID‐19 pandemic, uncontrolled forest/bushfires, and exposure to outdoor air pollution have contributed to an increased incidence and exacerbation of respiratory diseases. Human‐relevant preclinical models of respiratory diseases are valuable in expediting pulmonary research and in developing pulmonary therapies at sustainable costs.^15^ The efficacy of preclinical models in predicting human drug responses is vital to avoid failures of expensive and lengthy clinical trials. Animal models are the mainstay of therapeutic research and provide valuable data on in vivo pharmacokinetics, responses, and efficacy, and are mandatory for preclinical evaluation and approval of any novel or repurposed drugs. The investigation of complex physiological processes, their interactions, inflammatory and immune responses, and disease pathogenesis at the tissue and organ level and multiorgan interconnections is not possible with conventional cell culture models and must be performed in animal models.^188^

Unfortunately, the cross‐species variation of animal models in lung physiology, underlying molecular and cellular mechanisms, and gene expressions with humans creates issues with the predictive power for human drug responses.^188^ Moreover, they are expensive, time‐consuming, and may have ethical issues.^231^ Organoids and organs‐on‐a‐chip are novel technologies with promising applications in organ development, disease modeling, and the physiology of human organs.^191^ Organoids replicate critical structural and functional in vivo properties, whereas the organ‐on‐a‐chip model provides reproducible results with precise control of the microenvironment and inclusion of biophysical and chemical cues. The combination of these two systems can create a state‐of‐the‐art personalized disease model using organoids derived from a patient's tissue specimen.^169^ Multiple organs‐on‐chips, each representing an organ of the human body, can be integrated to develop multiorgan systems or body‐on‐chips that provide an additional prediction of drug responses in the human body.^231^ However, it is worth noting that lung organoids, lung‐on‐a‐chip, or lung organoids‐on‐a‐chip do not mimic the function of the whole lung; instead, they represent a major functional subunit of the lungs. They should be used as complementary methods with in vivo animal models to provide greater predictive power particularly of studies of pathogenesis and therapies in different organs. Recent advances in technology and refinement of existing models have led to significant progress in preclinical testing models. A definitive preclinical model that addresses all the issues of translational pulmonary is elusive. All models fundamentally complement each other, each assisting in drug development processes. Thus, the selection of screening models at each stage of the drug development process is vital to successfully define the most effective and safest drug in the market at a reduced cost.

## CONFLICT OF INTEREST STATEMENT

The authors declare no conflict of interest.

## Data Availability

Data sharing not applicable to this article as no data sets were generated or analyzed during the current study.
